# Vitamin D status during and after treatment and ovarian cancer survival

**DOI:** 10.1007/s10552-023-01757-0

**Published:** 2023-08-01

**Authors:** Tanya L. Ross, Rachel E. Neale, Renhua Na, Penelope M. Webb, for the OPAL Study Group

**Affiliations:** 1 Population Health Program, QIMR Berghofer Medical Research Institute, Brisbane, Australia; 2 School of Public Health, The University of Queensland, Brisbane, Australia

**Keywords:** Vitamin D, Ovarian cancer, Survival

## Abstract

**Purpose:**

Five-year relative survival for ovarian cancer remains below 50%. Strategies to improve outcomes are needed. Higher serum 25-hydroxyvitamin D [25(OH)D] concentrations [measure of vitamin D status] at and before diagnosis have been associated with longer survival in cancer patients; however, data for ovarian cancer are limited. We aimed to determine if 25(OH)D concentrations during and after primary treatment were associated with ovarian cancer-specific survival.

**Methods:**

We used data from a nationwide prospective cohort study of women with ovarian cancer. Among 886 participants treated with chemotherapy, 700 (79%) had a blood sample collected during (*n* = 591) and/or after (*n* = 458) primary treatment. These were tested for 25(OH)D. Clinical and survival data were abstracted from medical records. We used multivariable Cox proportional hazards regression to estimate hazard ratios (HR) and 95% confidence intervals (CI) for associations between 25(OH)D and ovarian cancer-specific survival.

**Results:**

Mean 25(OH)D concentrations were lower during than after primary treatment (82 and 91 nmol/L, respectively); only 14% and 8% had concentrations below 50 nmol/L during and after primary treatment, respectively. There was no association between 25(OH)D and ovarian cancer-specific survival during five years of follow-up [HR 1.10 (95% CI: 0.76, 1.61) and 0.95 (0.54, 1.68) for the highest vs. lowest quintile during and after treatment, respectively].

**Conclusions:**

We did not observe any association between serum 25(OH)D concentration and ovarian cancer-specific survival. Our results suggest that, in the absence of vitamin D deficiency, vitamin D supplementation to improve ovarian cancer survival is not warranted.

**Supplementary Information:**

The online version contains supplementary material available at 10.1007/s10552-023-01757-0.

## Introduction

Ovarian cancer is the eight most common incident cancer and seventh most common cause of cancer death in the female population worldwide [1]. More than two-thirds of those affected are diagnosed with advanced disease [2] and while the majority of patients respond to primary treatment, most will relapse. Despite a modest improvement over the last 25 years, 5-year relative survival remains below 50% in Australia and other high income countries [3–5].

When bound to the active form of vitamin D, the vitamin D receptor (VDR) plays a role in regulating the expression of more than 900 genes, which are involved in calcium homeostasis, control of cell cycling and growth, cellular differentiation, and immune response [6]. High expression of VDR has been seen in ovarian cancer cells [7, 8], and the active form of vitamin D has been shown to inhibit cell proliferation and induce apoptosis in ovarian cancer cells in vitro [7, 9], although at much higher concentrations than occur naturally. Furthermore, platinum and taxane agents showed greater inhibition of ovarian cancer cell proliferation with the addition of high concentrations of active vitamin D [7]. This suggests vitamin D may improve the response to chemotherapy in ovarian cancer.

Randomised controlled trials (RCTs) of vitamin D supplementation and cancer mortality have had mixed results, with two recent meta-analyses finding no significant association with cancer mortality overall [10, 11], although a benefit was seen for lung cancer mortality [10] and in the subset of trials that used daily dosing [11]. Neither meta-analysis reported ovarian cancer mortality specifically, nor have any trials examined cancer survival. A meta-analysis of prospective cohort studies found higher concentrations of 25(OH)D (presumably pre-diagnosis) were associated with reduced cancer mortality [12]. This meta-analysis did not look at the association by cancer site, but did find a stronger association among the female population. A more recent observational study found higher pre-diagnosis 25(OH)D concentrations were associated with improved overall cancer survival; however, in site-specific analysis a significant benefit was only seen for lung cancer with no association in the small (*n* = 74) subgroup with ovarian cancer [13]. In contrast, 25(OH)D concentrations less than 50 nmol/L at diagnosis, but not after treatment, were associated with poorer survival in one ovarian cancer study (*n* = 670 at diagnosis and *n* = 336 after treatment) [14].

If a survival benefit with higher 25(OH)D concentration is confirmed, vitamin D supplementation could be recommended for ovarian cancer patients. The period during primary treatment is of particular relevance as this is when 25(OH)D concentrations are likely to be lowest due to increased time indoors recovering from surgery and receiving chemotherapy, and in vitro studies have suggested 25(OH)D may have an additive benefit to chemotherapy [7]. We, therefore, aimed to determine if serum 25(OH)D concentrations: (i) during; and (ii) after primary treatment are associated with survival in women with ovarian cancer. We hypothesised that low 25(OH)D concentrations would be associated with poorer survival.

## Methods

### Participants

The Ovarian Cancer, Prognosis and Lifestyle (OPAL) study is a national prospective cohort study of 958 Australian women aged 18–79 years with a diagnosis of primary invasive epithelial ovarian, peritoneal or fallopian tube cancer between January 2012 and April 2015. Participants were identified through major treatment centres and approached as soon as possible after diagnosis. They were asked to complete questionnaires at recruitment (T0), then at three-monthly intervals for the first year after diagnosis (T3, T6, T9, T12), then annually (T24, T36, T48). From these we obtained sociodemographic (T0), medical and lifestyle data. Clinical data, treatment information, disease recurrence and vital status were abstracted annually from women’s medical records. Blood samples were collected at recruitment (median 2 and 3 months after the start of primary treatment for those undergoing adjuvant and neoadjuvant treatment, respectively), and again at 12 months after diagnosis if women were recurrence-free (median 8 and 6 months after the end of primary treatment for adjuvant and neoadjuvant treatment, respectively). Approval was obtained from the Human Research Ethics Committees of QIMR Berghofer Medical Research Institute and all participating centres. Participants provided informed consent.

**Fig. 1:**
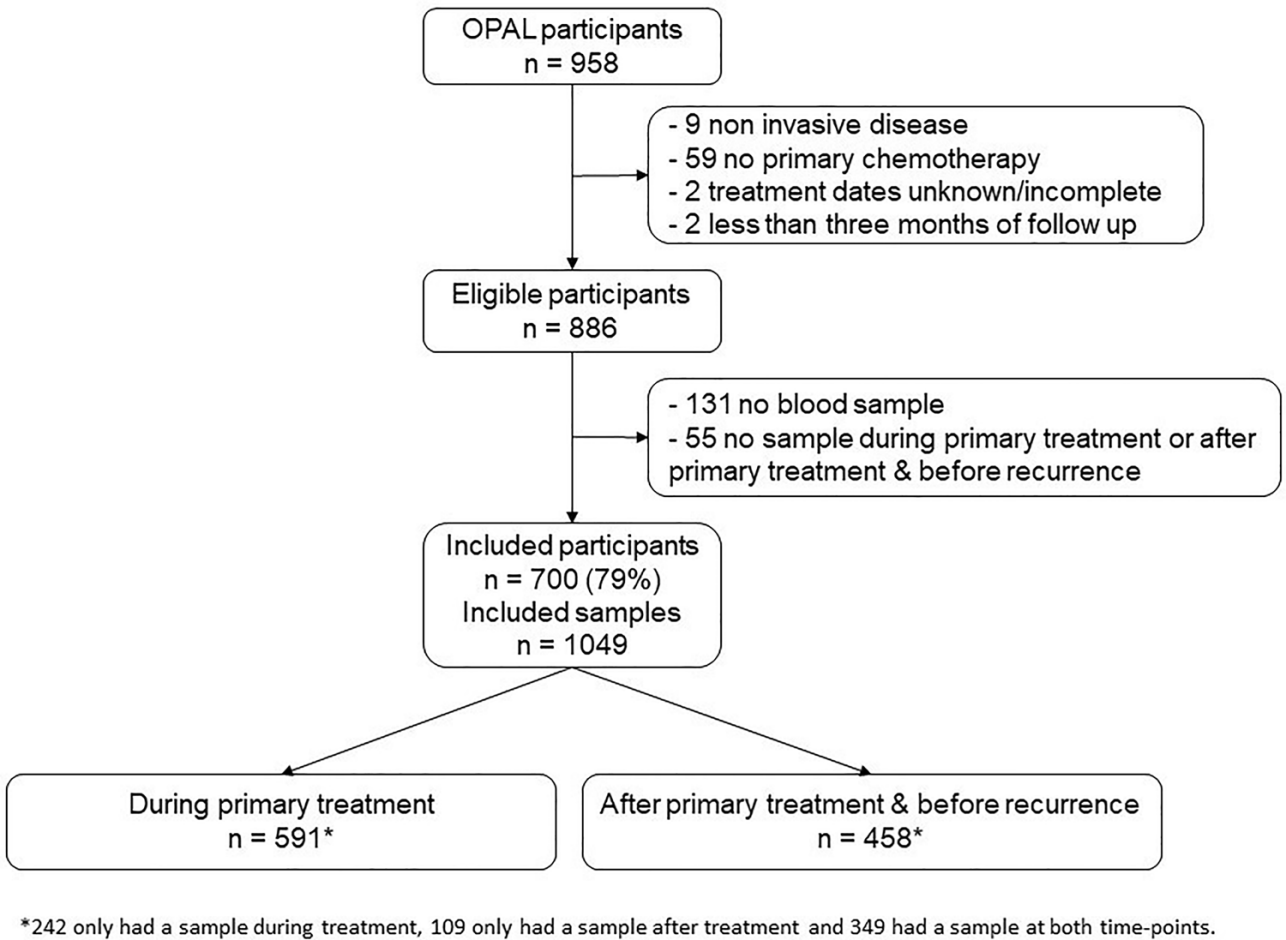
Flow diagram of participants and sample inclusion

Figure 1 shows the flow of participants through to analysis. Only participants with invasive disease who underwent chemotherapy as part of primary treatment, had recorded dates for initiation and completion of primary treatment, and at least 3 months of follow-up data were eligible for inclusion (*n* = 886, Fig. 1). We considered blood samples to be ‘during’ primary treatment if they were collected any time between the start of any treatment to within 30 days of finishing primary treatment. Samples were considered ‘after’ primary treatment if collected 31 days to 18 months after finishing primary treatment (and before recurrence). We excluded samples taken after recurrence or progression from the ‘after’ primary treatment analysis to avoid including those collected while the participant was on active treatment. The mean time from the start of treatment to collection of bloods was 2.5 months (SD 1.5) and 12.6 months (SD 2.3) for samples classified as ‘during’ and ‘after’ primary treatment, respectively. Participants who did not provide a blood sample (*n* = 131), and those that did not have a sample suitable for testing collected during or after primary treatment as defined above (*n* = 55) were excluded. Of the 886 eligible participants, 700 (79%) had one (351) or two (349) eligible blood samples, with 591 samples collected during primary treatment; and 458 samples collected after primary treatment and before recurrence.

### 25(OH)D concentrations

Samples were tested by a laboratory involved in the international Vitamin D Standardization Program using liquid chromatography tandem mass spectroscopy [15]. This measures 25(OH)D_2_ (ergocalciferol) and 25(OH)D_3_ (cholecalciferol). Total 25(OH)D (ergocalciferol + cholecalciferol) was used for this analysis. As there is notable seasonal variation in 25(OH)D concentrations we used a previously described technique to deseasonalize measures [16]; we did this separately for the samples collected during and after treatment as mean 25(OH)D differed between the two groups. 25(OH)D concentrations of 50 nmol/L or more are considered sufficient according to current guidelines; however, this cut-point was derived using older immunoassays and may not apply to newer assays such as that used here, which tends to give higher values. We thus categorised the deseasonalized 25(OH)D concentrations into quintiles for the primary analysis. For comparison with other studies we also created the following categories: less than 50 nmol/L; 50–74.9 nmol/L; 75–99.9 nmol/L; and 100 nmol/L or more.

### Survival

Survival was measured from the start of treatment to the latest date a woman was last known to be alive or truncated at five years after the start of primary treatment. Only 12 individuals were lost to follow-up during this five-year interval [their mean follow-up was 45.4 months (range 24.0 – 59.8)]. We assessed ovarian cancer-specific survival (OCS) to exclude any effect vitamin D may have on all-cause mortality. Those who died from other causes (*n* = 6, 1%) were censored at the time of death.

### Statistical analysis

We used Cox proportional hazards regression models to calculate hazard ratios (HR) and 95% confidence intervals (95% CI) for the association between 25(OH)D (i) during and (ii) after primary treatment, and ovarian cancer-specific survival. Survival time was left-truncated to the date of blood collection to avoid immortal time bias. Covariate inclusion was informed by a directed acyclic graph (DAG) and linear regression to identify factors associated with deseasonalized 25(OH)D in the included population (Supplementary Table 1). As not all lifestyle information was collected at every questionnaire time-point, and some participants were recruited several months after diagnosis and missed some questionnaires, up to 25% of participants were missing measures of current physical activity, time spent outdoors or vitamin supplementation around the time of blood collection. As these factors did not appreciably alter the estimates of association between 25(OH)D and OCS in complete case analyses, they were not included in the final models. All models were adjusted for age at diagnosis and conditioned on FIGO stage of disease at diagnosis to allow the baseline hazard to vary by stage. Fully-adjusted models were additionally adjusted for body mass index (BMI) (< 25 kg/m^2^, 25–29.9 kg/m^2^, ≥ 30 kg/m^2^) 5 years prior to diagnosis, smoking status at the time of recruitment (ever versus never smokers), and Charlson comorbidity index [17] based on self-reported medical conditions prior to diagnosis (0, 1, ≥ 2). Proportional hazards assumptions testing indicated the association between smoking status and OCS varied slightly over time; however, inclusion of an interaction term for smoking status × time did not alter the magnitude of the associations between 25(OH)D and survival, and this was therefore omitted from the final models.

We also analysed the association between serum 25(OH)D concentration during treatment and ovarian cancer survival including all eligible participants (*n* = 886) using multiple imputation to impute missing 25(OH)D quintile (33%) and covariate data (7%). Those missing a blood sample on treatment were slightly more likely to be of Asian ethnicity (9 vs. 4%), have 2 or more significant comorbidities (14 vs. 8%) and a low physical activity index prior to diagnosis (54 vs. 46%) than those with blood samples, but were similar in other characteristics. Under the assumption of missing at random, we used the multivariate imputation by chained equations (MICE) method [18], and included the previously identified covariates and the following auxiliary variables in the imputation model: ethnicity, education, FIGO stage and histotype at diagnosis, season of blood collection, deseasonalized 25(OH)D quintile, physical activity, time spent outdoors, any supplementation and vitamin D supplementation at or around the time of blood collection, and the cumulative baseline cause-specific hazard and binary indicator variable for the outcome [19, 20]. We set the time of blood collection for imputed values to the median time among those with samples (2 and 3 months after the start of primary treatment for those receiving adjuvant and neoadjuvant treatment, respectively). We imputed 20 datasets and combined the resulting estimates and standard errors using Rubin’s rules [21].

Ethnicity may also affect 25(OH)D and survival (via histotype), but as 89% of the included population were of white ethnicity this was not included in models. We conducted a subgroup analysis to determine if the association was the same when only those of white ethnicity were included. Further analyses were restricted to those: (i) with FIGO stage III and IV disease; (ii) who had cytoreductive surgery as part of treatment; and (iii) taking less than 500 IU/day of supplemental vitamin D daily around the time of blood collection.

Imputation and analysis of imputed data was completed using R statistical software (version 4.1.3, R Development Core Team, 2022). Other analyses were conducted using SAS version 9.4 (SAS Institute, Cary, North Carolina).

## Results

The mean age of included participants (*n* = 700) at diagnosis was 60 years, 75% had advanced stage disease, and 77% had high-grade serous carcinoma (Supplementary Table 2). 22% were taking daily vitamin D supplements of at least 500IU (12.5 micrograms cholecalciferol, indicative of regular supplementation) prior to diagnosis (Supplementary Table 2). Eligible participants who were excluded were slightly older (mean age 62 vs. 60 years), and more likely to be of Asian ethnicity (10 vs. 4%), have multiple comorbidities (16 vs. 8%), be current smokers (10 vs. 4%), and to have received chemotherapy only (9 vs. 3%), than those included (Supplementary Table 2). Otherwise the excluded women were very similar. The demographic and clinical characteristics of participants included in the analyses of 25(OH)D during and after primary treatment were very similar, except slightly fewer had primary surgery and adjuvant chemotherapy (65 vs. 72%) in the during-treatment analysis (Table 1).

**Table 1:** Demographic and clinical characteristics of included participants

	During primary treatment (*n* = 591^a^)		After primary treatment (*n* = 458^a^)	
	*n*	(%)	*n*	(%)
Age at diagnosis (years), mean (SD)	60	(11)	60	(10)
Ethnicity				
White	523	(89)	405	(89)
Asian	23	(4)	18	(4)
All others and mixed	42	(7)	33	(7)
Highest education level				
School age 15–16 or less	217	(37)	164	(36)
School age 17–18 or diploma/trade certificate	215	(37)	164	(36)
University	157	(27)	130	(28)
Charlson comorbidity index^b^				
0	439	(75)	350	(76)
1	102	(17)	78	(17)
≥ 2	48	(8)	30	(7)
Smoking status at recruitment				
Never smoker	317	(54)	253	(55)
Ex-smoker	249	(42)	190	(41)
Current smoker	24	(4)	15	(3)
BMI before diagnosis (kg/m^2^)				
< 25	254	(43)	187	(41)
25 to 29.9	196	(33)	168	(37)
≥ 30	141	(24)	103	(22)
Physical activity before diagnosis				
Low	231	(46)	175	(46)
Moderate	77	(15)	53	(14)
High	198	(39)	152	(40)
Time spent outdoors before diagnosis (hours/week), mean (SD)	8.4	(6.8)	8.6	(6.9)
Vitamin D supplement use before diagnosis				
Nil	283	(56)	214	(57)
< 500 IU/day	112	(22)	80	(21)
≥ 500 IU/day	111	(22)	82	(22)
FIGO stage at diagnosis				
I	92	(16)	90	(20)
II	59	(10)	56	(12)
III	361	(61)	267	(58)
IV	79	(13)	45	(10)
Histology				
High-grade serous	457	(77)	344	(75)
Mucinous	13	(2)	15	(3)
Endometrioid	38	(6)	38	(8)
Clear cell	35	(6)	26	(6)
Low-grade serous	14	(2)	11	(2)
Carcinosarcoma/mixed/other	34	(6)	24	(5)
Primary treatment				
Primary cytoreductive surgery + adjuvant chemotherapy	387	(65)	330	(72)
Neoadjuvant chemotherapy + interval cytoreductive surgery	183	(31)	125	(27)
Chemotherapy, no cytoreductive surgery^c^	21	(5)	3	(1)

^a^349 participants are included in both during primary treatment and after primary treatment analyses. Numbers may not sum to total because of missing data

^b^Charlson comorbidity index score determined using self-report of conditions diagnosed by a doctor prior to diagnosis

^c^The majority of individuals that did not have cytoreduction had disease that was not resectable or a contraindication to surgery

Mean deseasonalized 25(OH)D concentrations were lower during primary treatment [82 nmol/L (SD 32 nmol/L)], than after treatment [91 nmol/L (SD 32 nmol/L)]. Only 14% and 8% of samples had concentrations below 50 nmol/L during and after primary treatment, respectively. Five-year OCS was 55% (260 ovarian cancer deaths) and 74% (118 ovarian cancer deaths) for participants included in during-treatment and after-treatment analyses, respectively (Table 2). This difference was expected, as women included in the after-treatment analysis had survived an average of 12.6 months without recurrence when their post-treatment bloods were collected. Mean follow-up time contributing to analyses for those alive at last contact was 59.6 months and 59.7 months for during-treatment and after-treatment analyses, respectively.

**Table 2:** Hazard ratios (HR) for the association between deseasonalized 25(OH)D concentration and ovarian cancer-specific survival (OCS) up to 5 years

Deseasonalized 25(OH)D quintiles (nmol/L)	OC deaths within 5 years/total	OCS at 5 years (%)	HR (95% CI)^a^	Fully adjusted HR (95% CI)^b^
During primary treatment (n = 589^c^); overall OCS at 5 years 55%				
Quintile 1 (7.6–54.1)	55/119	53	Referent	Referent
Quintile 2 (54.2–70.7)	51/118	56	1.01 (0.69, 1.48)	1.01 (0.69, 1.49)
Quintile 3 (70.8–89.1)	52/117	55	1.09 (0.74, 1.59)	1.07 (0.73, 1.57)
Quintile 4 (89.2-105.8)	46/118	60	0.76 (0.51, 1.12)	0.72 (0.48, 1.07)
Quintile 5 (105.9-307.9)	56/117	52	1.18 (0.81, 1.71)	1.10 (0.76, 1.61)
After primary treatment (n = 458); overall OCS at 5 years 74%^d^				
Quintile 1 (14.1–65.6)	23/92	75	Referent	Referent
Quintile 2 (65.7–81.3)	26/92	71	1.18 (0.67, 2.07)	1.27 (0.72, 2.23)
Quintile 3 (81.4–95.9)	22/90	75	1.01 (0.56, 1.82)	1.00 (0.56, 1.81)
Quintile 4 (96.0-113.7)	20/92	78	0.93 (0.51, 1.69)	1.00 (0.54, 1.83)
Quintile 5 (113.8-275.9)	27/92	70	1.10 (0.63, 1.92)	0.95 (0.54, 1.68)

*OC* ovarian cancer

^a^Stratified by FIGO stage and adjusted for age

^b^Stratified by FIGO stage and adjusted for age at diagnosis, smoking status (ever vs. never) at recruitment, BMI category (< 25 kg/m, 25–29.9 kg/m^2^, ≥ 30 kg/m^2^) and Charlson comorbidity index (nil, 1, ≥ 2) prior to diagnosis

^c^Excludes 2 participants missing covariate data

^d^Individuals with recurrence before collection of second blood sample (~ 12 months) were excluded from ‘after primary treatment’ analysis

Table 2 shows the results of the main analysis. While the HR was below one among those in the fourth quintile of 25(OH)D during treatment (0.72; 95% CI: 0.48, 1.07) compared to the lowest quintile, the HRs for the other quintiles were all above one and there was no linear trend with increasing 25(OH)D. Analysis using imputed 25(OH)D quintiles for those missing blood samples during primary treatment did not change the results (Supplementary Table 3).

There was no association between 25(OH)D and OCS in analysis of 25(OH)D concentration measured after treatment (HR 0.95; 95% CI: 0.54, 1.68; highest compared to lowest quintile) (Table 2). There was no increase in ovarian cancer mortality in those with concentrations below 50 nmol/L in during-treatment or after-treatment analyses (Supplementary Table 4).

When we excluded those taking 500 IU or more of vitamin D supplementation, those with 25(OH)D concentrations in the fourth quintile during primary treatment (89.2–105.8 nmol/L) appeared to have better survival (HR 0.60; 95% CI: 0.34–1.07) compared to those in the lowest quintile; however, this was not seen in the highest quintile and there was no trend across quintiles (Table 3). After primary treatment, the HR for quintiles two to five compared to the lowest quintile decreased with increasing 25(OH)D; however, they were all over 1.0, except quintile five (HR: 0.85; 95% CI: 0.40, 1.80) (Table 3). The results were essentially unchanged when we restricted the population to white ethnicity, FIGO stage III and IV disease, and those that received cytoreductive surgery (Table 3).

**Table 3:** Fully adjusted hazard ratios (HR) for the association between deseasonalized 25(OH)D concentration and ovarian cancer-specific survival (OCS) up to 5 years, subgroup analyses

Deseasonalized 25(OH)D quintiles (nmol/L)	White ethnicity only HR (95%CI)^a^	Stage III/IV only HR (95%CI)^a^	Only those that had cytoreductive surgery HR (95%CI)^a^	Excluding those taking ≥ 500IU vitamin D supplement daily HR (95%CI)^a^
During primary treatment	*n* = 521	*n* = 438	*n* = 568	*n* = 349
Quintile 1 (7.6–54.1)	Referent	Referent	Referent	Referent
Quintile 2 (54.2–70.7)	1.04 (0.69, 1.57)	1.03 (0.69, 1.53)	0.96 (0.64, 1.43)	1.14 (0.71, 1.82)
Quintile 3 (70.8–89.1)	1.05 (0.70, 1.59)	1.09 (0.73 1.62)	1.03 (0.69, 1.53)	1.29 (0.79, 2.10)
Quintile 4 (89.2-105.8)	0.72 (0.47, 1.11)	0.69 (0.45, 1.04)	0.71 (0.47, 1.07)	0.60 (0.33, 1.06)
Quintile 5 (105.9-307.9)	1.15 (0.77, 1.73)	1.13 (0.76, 1.67)	1.07 (0.72, 1.59)	1.06 (0.63, 1.80)
After primary treatment	*n* = 405	*n* = 312	*n* = 455	*n* = 291
Quintile 1 (14.1–65.6)	Referent	Referent	Referent	Referent
Quintile 2 (65.7–81.3)	1.20 (0.66, 2.18)	1.28 (0.70, 2.34)	1.28 (0.72, 2.26)	1.45 (0.77, 2.73)
Quintile 3 (81.4–95.9)	1.07 (0.58, 1.96)	1.06 (0.57, 1.96)	1.01 (0.56, 1.83)	1.11 (0.55, 2.24)
Quintile 4 (96.0-113.7)	0.91 (0.48, 1.74)	1.09 (0.58, 1.96)	0.97 (0.52, 1.80)	1.08 (0.52, 2.26)
Quintile 5 (113.8-275.9)	0.96 (0.53, 1.75)	0.97 (0.53, 1.77)	0.95 (0.53, 1.69)	0.85 (0.40, 1.80)

^a^Stratified by FIGO stage and adjusted for age at diagnosis, smoking status (ever vs. never) at recruitment, BMI category (< 25 kg/m, 25–29.9 kg/m^2^, ≥ 30 kg/m^2^) and Charlson comorbidity index (nil, 1, ≥ 2) prior to diagnosis

## Discussion

We did not find evidence of an association between 25(OH)D concentrations in blood samples collected during or after primary treatment and OCS in this Australian population. However, vitamin D deficiency [currently defined as 25(OH)D < 50 nmol/L] was comparatively rare; thus we had limited power to detect an association between vitamin D deficiency and survival.

To the best of our knowledge, no other study has examined 25(OH)D concentrations during treatment and ovarian cancer survival, and only one other has used measures from samples collected after treatment. Our after-treatment results are consistent with a previous Australian study that did not find a significant association between 25(OH)D concentrations after treatment and survival [14]. While not directly comparable, our during-treatment results are consistent with a previous small study (*n* = 74) that found no association between pre-diagnosis serum 25(OH)D concentrations and ovarian cancer survival (HR 0.93; 95% CI: 0.41, 2.09; highest vs. lowest tertile) [13]. However, they are not consistent with the earlier Australian study that found lower 25(OH)D concentrations at diagnosis were associated with poorer survival in ovarian cancer (< 25 nmol/L HR 1.44; 95% CI: 1.07, 1.95 and 25–49.9 nmol/L HR 1.30; 95% CI: 1.01, 1.66; compared to 50.0–74.9 nmol/L) [14]. One other small study (*n* = 72) also found ovarian cancer patients with low (< 25 nmol/L) 25(OH)D before primary treatment had poorer survival compared to those with concentrations of 25 nmol/L or more; however that analysis was not adjusted for confounders [22]. The differing timing of sampling of these studies may have contributed to the differences in results.

In both previous studies that found lower 25(OH)D concentrations were associated with poorer survival in ovarian cancer [14, 22], vitamin D deficiency was much more common. It is plausible that this difference is at least partly due to the different methods used to measure 25(OH)D. However, the use of vitamin D supplementation (≥ 500 IU daily) has increased in the ten years between the first Australian study (< 1%; personal communication) and this study (22%), so it is also likely that there are true differences in 25(OH)D concentrations between the two.

It is possible that any association between 25(OH)D and cancer survival may be affected by genetic factors. If this were the case, then genetic differences in study populations could contribute to inter-study differences. A recent study observed improved overall cancer and lung cancer survival in those with higher pre-diagnosis 25(OH)D concentrations, but only among those with particular vitamin D binding protein (VDBP) isoforms [13]. Polymorphisms in the VDR may also influence associations between 25(OH)D and cancer survival. An association between VDR polymorphisms and survival in ovarian cancer patients has previously been observed in a small study (*n* = 101) [23]. Further exploration of how VDBP and VDR polymorphisms may influence the relationship between 25(OH)D concentrations and survival is warranted.

A strength of our study is the use of a gold-standard measure of 25(OH)D, which is likely to be more accurate than measures used in older studies [15]. As samples were collected at different times of the year we also used a technique to deseasonalize 25(OH)D concentrations to reduce the risk of misclassifying overall vitamin D status, particularly if a sample had been collected at the end of winter or summer. However, our study was limited by low power to detect an association between vitamin D deficiency and survival as only a small proportion of the study population had 25(OH)D concentrations less than 50 nmol/L.

## Conclusion

Our results do not support an association between 25(OH)D concentrations and OCS at the population level and do not replicate a trend towards better survival in those with higher 25(OH)D concentrations seen in a previous cohort. Our results do not support universal vitamin D supplementation of ovarian cancer patients in a largely vitamin D sufficient population such as Australia.

### Supplementary Information

Below is the link to the electronic supplementary material.

Supplementary file1 (DOCX 33 kb)

## Data Availability

The datasets generated during and/or analysed during the current study are not available as open access as participants did not consent to this.
