# Supplementary material for: Vitamin D status during and after treatment and ovarian cancer survival
Source: Cancer Causes Control. 2023 Aug 1;35(1):1–8. doi: 10.1007/s10552-023-01757-0 (PMC10764528; doi:10.1007/s10552-023-01757-0)
Supplement: Supplementary file 1 — Supplementary file1 (DOCX 33 kb) [file 10552_2023_1757_MOESM1_ESM.docx]

**Supplementary material: Vitamin D status during and after treatment and ovarian cancer survival**

Cancer Causes & Control

Authors: Tanya L. Ross^1,2^, Rachel E. Neale^1,2^, Renhua Na^1,2^, Penelope M. Webb^1,2^ for the OPAL Study Group

^1^ Population Health Program, QIMR Berghofer Medical Research Institute, Brisbane, Australia

^2^ School of Public Health, The University of Queensland, Brisbane, Australia

Corresponding author: Tanya L. Ross ([Tanya.Ross@qimrberghofer.edu.au](mailto:Tanya.Ross@qimrberghofer.edu.au))

**Supplementary Table 1: Age-adjusted mean^a^ deseasonalized 25(OH)D concentration by demographic and clinical characteristics.**

|  | **During treatment**  **n = 591** |  | **After treatment**  **n=458** |  |
| --- | --- | --- | --- | --- |
|  | **nmol/L (95%CI)** | **p** | **nmol/L (95% CI)** | **p** |
| Age at diagnosis |  |  |  |  |
| <50 years | 78.9 (72.7, 85.0) | 0.3 | 96.3 (89.6, 103) | 0.2 |
| 50-59 years | 81.5 (76.5, 86.5) |  | 88.6 (83.1, 94.1) |  |
| 60-69 years | 80.6 (76.3, 84.9) |  | 92.4 (87.6, 97.3) |  |
| 70-79 years | 86.0 (80.0, 91.9) |  | 86.5 (79.4, 93.6) |  |
| *Per year* | 0.14 (-0.06, 0.43) | 0.1 | -0.11 (-0.40,0.18) | 0.5 |
| Ethnicity |  |  |  |  |
| White | 82.6 (79.9, 85.3) | 0.02 | 92.3 (89.2, 95.4) | 0.02 |
| All others and mixed combined | 73.0 (65.2, 80.8) |  | 80.9 (72.1, 89.8) |  |
| Highest education level |  |  |  |  |
| School age 15-16 or less | 82.7 (78.3, 87.1) | 0.8 | 92.5 (87.5, 97.6) | 0.1 |
| School age 17-18/dip./trade cert. | 80.4 (76.1, 84.7) |  | 87.1 (82.2, 92.0) |  |
| University | 81.3 (76.1, 86.5) |  | 94.3 (88.6, 100) |  |
| Charlson comorbidity index^b^ |  |  |  |  |
| 0 | 81.5 (78.5, 84.5) | 0.98 | 90.2 (86.9, 93.6) | 0.4 |
| 1 | 81.1 (74.8, 87.3) |  | 95.5 (88.4, 103) |  |
| ≥2 | 82.3 (73.1, 91.5) |  | 89.6 (78.0, 101) |  |
| Smoking status at recruitment |  |  |  |  |
| Never smoker | 79.3 (75.8, 82.9) | 0.2 | 91.0 (87.1, 95.0) | 0.07 |
| Ex-smoker | 84.3 (80.3, 88.3) |  | 92.6 (88.1, 97.1) |  |
| Current smoker | 81.2 (68.3, 94.0) |  | 73.1 (56.9, 89.2) |  |
| BMI before diagnosis (kg/m^2^) |  |  |  |  |
| <25 | 86.4 (82.5, 90.4) | 0.005 | 96.4 (91.8, 101) | 0.005 |
| 25 to 29.9 | 78.5 (74.0, 83.0) |  | 89.5 (84.6, 94.3) |  |
| ≥30 | 77.1 (71.8, 82.3) |  | 84.1 (78.0, 90.3) |  |
| *Per kg/m^2^* | -0.85 (-1.29, -0.41) | 0.0002 | -1.24 (-1.78, -0.70) | <0.0001 |
| Physical activity (per MET hour/week)^c^ | 0.07 (-0.15, 0.30) | 0.5 | 0.11 (-0.03, 0.24) | 0.1 |
| Outside time (per hour/week)^c^ | 0.82 (0.35, 1.29) | 0.0007 | 0.18 (-0.21, 0.50) | 0.4 |
| Any supplement use ^c^ |  |  |  |  |
| No | 73.4 (69.0, 77.9) | <0.0001 | 84.4 (78.7, 90.1) | 0.006 |
| Yes | 88.4 (84.5, 92.3) |  | 94.5 (90.9, 98.0) |  |
| Unknown | 80.6 (75.4, 85.7) |  | 84.6 (73.9, 95.2) |  |
| Vitamin D supplementation^c^ |  |  |  |  |
| No | 74.8 (71.2, 78.5) | <0.0001 | 83.3 (79.3, 87.4) | <0.0001 |
| Less than 500IU/day | 86.4 (79.4, 93.5) |  | 91.2 (84.1, 98.2) |  |
| 500IU or more | 98.7 (92.5, 105) |  | 106 (100, 111) |  |
| Unknown | 80.4 (75.3, 85.4) |  | 84.5 (74.2, 94.8) |  |
| FIGO stage at diagnosis |  |  |  |  |
| I | 84.9 (78.3, 91.5) | 0.4 | 86.2 (79.5, 92.9) | 0.4 |
| II | 78.9 (70.7, 87.1) |  | 90.7 (82.3, 99.1) |  |
| III | 80.3 (77.0, 83.7) |  | 92.2 (88.4, 96.1) |  |
| IV | 85.3 (78.1, 92.4) |  | 94.6 (85.3, 104) |  |
| Histology |  |  |  |  |
| High-grade serous | 81.0 (78.1, 84.0) | 0.5 | 92.8 (89.4, 96.2) | 0.06 |
| Other | 83.4 (77.9, 89.0) |  | 86.0 (80.0, 92.0) |  |
| Primary treatment |  |  |  |  |
| PCS + adjuvant CT | 80.2 (77.0, 83.5) | 0.2 | 89.6 (86.1, 93.1) | 0.1 |
| Neoadjuvant CT +/- ICS | 84.1 (79.6, 88.6) |  | 94.9 (89.3, 101) |  |

CT, chemotherapy; PCS, primary cytoreductive surgery; ICS, interval cytoreductive surgery

^a^Mean 25(OH)D for categorical variables and difference in 25(OH)D for continuous variables

^b^Charlson comorbidity index score determined using self-report of conditions diagnosed by a doctor

^c^Measured around the time of blood collection

**Supplementary table 2: Demographic and clinical characteristics of included versus excluded eligible participants.**

|  | **Included (N=700)^a^** | | **Eligible, but excluded (N=186)^a^** | |
| --- | --- | --- | --- | --- |
|  | **n** | **(%)** | **n** | **(%)** |
| Age at diagnosis (years), mean (SD) | 60 | (11) | 62 | (10) |
| Ethnicity |  |  |  |  |
| White | 620 | (89) | 146 | (82) |
| Asian | 30 | (4) | 18 | (10) |
| All others and mixed | 47 | (7) | 13 | (7) |
| Highest education level |  |  |  |  |
| School age 15-16 or less | 253 | (36) | 76 | (43) |
| School age 17-18 or diploma/trade certificate | 253 | (36) | 51 | (29) |
| University | 192 | (28) | 48 | (27) |
| Charlson comorbidity index^b^ |  |  |  |  |
| 0 | 523 | (75) | 124 | (67) |
| 1 | 117 | (17) | 30 | (16) |
| ≥2 | 58 | (8) | 30 | (16) |
| Smoking status at recruitment |  |  |  |  |
| Never smoker | 381 | (55) | 96 | (52) |
| Ex-smoker | 292 | (42) | 70 | (38) |
| Current smoker | 26 | (4) | 19 | (10) |
| BMI before diagnosis (kg/m^2^) |  |  |  |  |
| <25 | 290 | (41) | 84 | (45) |
| 25 to 29.9 | 238 | (34) | 56 | (30) |
| ≥30 | 172 | (25) | 45 | (24) |
| Physical activity before diagnosis |  |  |  |  |
| Low | 268 | (46) | 71 | (55) |
| Moderate | 82 | (14) | 19 | (15) |
| High | 227 | (39) | 38 | (30) |
| Time spent outdoors before diagnosis (hours/week), mean (SD) | 8.3 | (6.6) | 8.7 | (7.6) |
| Vitamin D supplementation before diagnosis |  |  |  |  |
| Nil | 322 | (56) | 57 | (48) |
| <500 IU / day | 125 | (22) | 34 | (29) |
| ≥500 IU / day | 126 | (22) | 27 | (23) |
| FIGO stage at diagnosis |  |  |  |  |
| I | 110 | (16) | 23 | (12) |
| II | 69 | (10) | 11 | (6) |
| III | 425 | (61) | 127 | (68) |
| IV | 96 | (14) | 25 | (13) |
| Histology |  |  |  |  |
| High-grade serous | 540 | (77) | 143 | (77) |
| Mucinous | 20 | (3) | 3 | (2) |
| Endometrioid | 46 | (7) | 18 | (10) |
| Clear cell | 38 | (5) | 8 | (4) |
| Low-grade serous | 17 | (2) | 5 | (3) |
| Carcinosarcoma/mixed/other | 39 | (6) | 9 | (5) |
| Primary treatment |  |  |  |  |
| Primary cytoreductive surgery + adjuvant chemotherapy | 460 | (66) | 110 | (59) |
| Neoadjuvant chemotherapy + interval cytoreductive surgery | 217 | (31) | 60 | (32) |
| Chemotherapy, no cytoreductive surgery^c^ | 23 | (3) | 16 | (9) |

^a^Numbers may not sum to total because of missing data

^b^Charlson comorbidity index score determined using self-report of conditions diagnosed by a doctor

^c^The majority of individuals that did not have cytoreduction had disease that was not resectable or a contraindication to surgery.

**Supplementary table 3: Hazard ratios (HR) for the association between quintiles of deseasonalized 25(OH)D concentrations during primary treatment (n=886) and ovarian cancer-specific survival (OCS) to 5 years from imputed^a^ analysis**

| **Deseasonalized 25(OH)D quintiles (nmol/L)** | **HR (95% CI)**^b^ | **Fully adjusted HR (95% CI)^c^** |
| --- | --- | --- |
| Quintile 1 (7.6-54.1) | Referent | Referent |
| Quintile 2 (54.2-70.7) | 1.01 (0.70, 1.45) | 1.03 (0.71, 1.49) |
| Quintile 3 (70.8-89.1) | 1.15 (0.81, 1.65) | 1.15 (0.80, 1.66) |
| Quintile 4 (89.2-105.8) | 0.78 (0.54, 1.12) | 0.73 (0.50, 1.06) |
| Quintile 5 (105.9-307.9) | 1.29 (0.92, 1.81) | 1.19 (0.84, 1.69) |

^a^25(OH)D quintiles imputed for those eligible but missing a blood sample during treatment (33%)

^b^Stratified by FIGO stage and adjusted for age

^c^Stratified by FIGO stage and adjusted for age at diagnosis, smoking status (ever vs never) at recruitment, BMI category (<25 kg/m, 25-29.9 kg/m^2^, ≥30 kg/m^2^) and Charlson comorbidity index (nil, 1, ≥2) prior to diagnosis.

**Supplementary table 4: Hazard ratios (HR) for the association between deseasonalized 25(OH)D concentration and ovarian cancer-specific survival (OCS) up to 5 years**

| **Deseasonalized 25(OH)D concentrations (nmol/L)** | **OC deaths within 5 years/total, n** | **OCS at 5 years** | **HR (95% CI)**^a^ | **Fully adjusted**  **HR (95% CI)^b^** |
| --- | --- | --- | --- | --- |
| ***During primary treatment (n=589^c^); overall OCS at 5 years 55%*** | | | |  |
| <50 | 39/82 | 52% | 1.08 (0.74, 1.58) | 1.04 (0.70, 1.53) |
| 50-74.9 | 82/185 | 55% | Referent | Referent |
| 75-99.9 | 72/164 | 56% | 0.95 (0.69, 1.30) | 0.90 (0.65, 1.24) |
| ≥100 | 67/158 | 57% | 0.89 (0.64, 1.23) | 0.82 (0.59, 1.13) |
| ***After primary treatment (n=458); overall OCS at 5 years 74%*** | | | |  |
| <50 | 7/36 | 80% | 0.76 (0.33, 1.74) | 0.62 (0.27, 1.43) |
| 50-74.9 | 30/100 | 69% | Referent | Referent |
| 75-99.9 | 37/159 | 77% | 0.83 (0.51, 1.36) | 0.77 (0.47, 1.26) |
| ≥100 | 44/163 | 73% | 0.91 (0.57, 1.46) | 0.79 (0.49, 1.28) |

^a^Stratified by FIGO stage and adjusted for age

^b^Stratified by FIGO stage and adjusted for age at diagnosis, smoking status (ever vs never) at recruitment, BMI category (<25 kg/m, 25-29.9 kg/m^2^, ≥30 kg/m^2^) and Charlson comorbidity index (nil, 1, ≥2) prior to diagnosis.

^c^Excludes 2 participants missing covariate data
